# Frontline love: Romantic partners of frontline doctors and nurses during the New York City COVID‐19 outbreak

**DOI:** 10.1111/jomf.12831

**Published:** 2022-03-10

**Authors:** Alana Siegel, Rachel Dekel

**Affiliations:** ^1^ Faculty of Humanities Tel Aviv University Tel Aviv Israel; ^2^ School of Social Work Bar‐Ilan University Ramat Gan Israel

**Keywords:** coronavirus, COVID‐19, crisis, doctor, Family Stress Model, frontline worker, healthcare worker, hospital, New York City, nurse, pandemic, partner, spouse

## Abstract

**Objective:**

This qualitative study's aim was to learn how the spouses and romantic partners of frontline doctors and nurses dealt with the acute stress of the outbreak; the kinds of support they provided when the frontliners had to navigate COVID‐19 at their hospitals; and, according to their perceptions, how this crisis impacted their relationship.

**Background:**

This study focused on the partners of frontliners working in hospitals during the crisis of the coronavirus outbreak in New York City (NYC)—one of the earliest epicenters of the COVID‐19 pandemic in the United States. This study expanded upon the Family Stress Model—which examines how economic problems can affect marital quality and stability.

**Method:**

Interviews were conducted with 29 partners of frontliners who had been treating COVID‐19 patients in NYC hospitals during the pandemic outbreak from February 29 to June 1, 2020. Partners were recruited via snowball sampling, interviewed via Zoom or telephone, and results were analyzed using thematic content analysis.

**Results:**

The following themes were found in the narratives: The burden of running the home independently; providing various kinds of support (concrete, emotional, and refraining from sexual and physical closeness); and the effects of the pandemic on the relationship via writing a will and discussing the possibility of death, the lack of relationship‐ending threats, and emerging from the crisis with a strengthened relationship.

**Conclusion:**

The pandemic crisis unified the partners and frontliners, even in the face of multiple stressors.

## INTRODUCTION

SARS‐CoV‐2, the highly contagious and novel coronavirus that causes COVID‐19, was discovered in Wuhan, China, in December 2019, and was declared a global pandemic by March 11, 2020 (World Health Organization, 2021). By March 22, enough virus cases had been found in New York City (NYC) that a citywide lockdown was declared (Ramachandran et al., 2020). Cases of COVID‐19 soon escalated, with 33,465 deaths in NYC from March 11 to May 11 (McFall‐Johnsen, 2020). In the pandemic's first 3 months, 203,000 cases were reported in NYC, leading the area to become one of the earliest virus epicenters in the United States. In this period, the fatality rate among confirmed cases in the city was 9.2%, and 32.1% among hospitalized patients (Thompson et al., 2020).

“Frontline workers” are defined as “employees within essential industries who must physically show up to their jobs” (Tomer & Kane, 2020). By the last week of March, NYC hospitals were overwhelmed, and lacked the following: beds for the overflow of patients, oxygen tanks and ventilators, and personal protective equipment (PPE) for frontline workers (Caspani, 2020). Frontliners reported wearing the same facemask for a week's time, reusing plastic gloves, and even donning garbage bags for protection (Bowden et al., 2020). In addition, countless healthcare workers were themselves falling sick with COVID‐19 (Schwirtz, 2020), resulting in hospital understaffing as they stayed home to convalesce, while other staff continued to work while sick or even died from the virus (Condon et al., 2020).

Frontliners were suddenly and unexpectedly faced with a virus with an unclear etiology and pathology, high numbers of sick and dying patients with no known cure, and the need to make ethical decisions for hundreds of critically ill patients—all while themselves fearing falling ill with COVID‐19 (Kisner, 2020). So many patients were simultaneously in distress or “coding” (i.e., experiencing cardiac arrest or a life‐threatening medical emergency in need of intervention) that healthcare workers were overwhelmed by whose life to save first (Cha et al., 2020), and they witnessed dozens of patient deaths daily (Brown & Borter, 2020). Frontline workers reported burnout (Chen et al., 2021), posttraumatic stress disorder (PTSD) (Gavin et al., 2020), anxiety, depression (The Physicians Foundation, 2020), hopelessness, and fatigue (Li et al., 2020) as a result of their work.

In addition to the struggles experienced at the hospital, frontline workers had to contend with an added level of grave concern: infecting their family upon returning home from work (Wu et al., 2020). Although many studies have focused on frontline workers, the current study focused on their partners, specifically on the way in which partners coped with and navigated the pandemic's outbreak. General family theories have suggested associations between work and home functioning (Dekel et al., 2016) as well as associations between each partner's functioning and mental distress in general (Hilpert et al., 2018), and in times of crisis in particular (Cohan et al., 2009). Moreover, given its clear relevance to the pandemic, we utilized the Family Stress Model (FSM), which examines how economic problems can affect marital quality and stability by decreasing the positive and increasing the negative behaviors that spouses demonstrate in their dyadic interactions (Conger et al., 1999).

Doctors and nurses had reason to be fearful of infecting their loved ones: They were indeed at higher risk for illness and transmission, and reported being deeply afraid of transmitting the virus to their families upon returning home from work (Miotto et al., 2020). Frontliners shared that upon returning home, they would change clothes, shower, and store their work clothes in closed bins until they could be laundered (Weise, 2020). However, it was subsequently understood that these practices were probably irrelevant, as the virus is airborne; in other words, family members may still have become infected despite frontliners engaging in these practices. In a study of healthcare workers in the United Kingdom, patient‐facing workers were found to have a threefold, and their household members a twofold, increased risk of hospital admission with COVID‐19 (Shah et al., 2020).

We could find only a paucity of literature on the current pandemic from the point of view of healthcare workers' spouses, and those studies that do exist mainly focus on spouses' distress, not on their coping. A few of these studies found that having a partner's support—as opposed to being single—was associated with better coping for the frontliner in the face of the virus (Du et al., 2020; Elbay et al., 2020). In a study from Ningbo, China, 845 family members of healthcare workers (65.4% of whom were spouses) reported a high level of anxiety (33.7%) and depression (29.35%) symptoms (Ying et al., 2020). Partners were confronted with the painful realization that their spouses could potentially die, possibly rapidly, from the virus (Bosman, 2020). Some spouses were shockingly faced with the loss of their frontliner partners upon their deaths from COVID‐19 (Gold, 2020). Recent studies on COVID‐19 have shown that, similar to their patients, frontliners have also had to navigate their fears of infection, stress, and the balancing of work and family (Madani, 2020). Just like the patients they treat, the shocking and sudden onset of the pandemic has been a major stressor for doctors and nurses, as well as for their partners and families (Arnetz et al., 2020; Wu et al., 2020). We were especially interested in the effects of an acute crisis on the relationships of those working on the frontlines of the COVID‐19 crisis in an early epicenter.

### The FSM and couples during a crisis

The FSM examines how economic problems can affect marital quality and stability by decreasing the positive and increasing the negative behaviors that spouses demonstrate in their dyadic interactions; for example, the couple's stress can spill over into challenges with parenting and increase interparental conflict (Conger et al., 1999). Specifically, the FSM hypothesizes that economic pressure (i.e., being unable to meet basic financial needs and the inability to pay bills) can lead to emotional distress, which can increase the risk for marital conflict and distress over time. However, high marital support can contribute to being less affected by economic pressure and subsequent emotional distress, and effective problem‐solving and supporting one another during times of stress can reduce the marital pressures a couple may encounter. Indeed, in the face of economic pressure, highly supportive couples have been found to experience less emotional distress than have couples with low levels of support (Conger et al., 1999). When confronting a stressor, it has been found that couples benefit from demonstrating sensitivity and concern, as well as engaging in bargaining, negotiating, agreement, and problem‐solving in order to best respond to marital conflict (Conger et al., 1999). The current study aimed to expand upon the FSM model, by focusing on what was happening in the relationship and the role of social support within a marriage during times of a major stressor, namely the COVID‐19 outbreak, which has included multiple stressors including economic and work instability.

The literature on romantic relationships has found different outcomes as to how couples face a crisis. Certain crises have resulted in increased divorce rates, such as in the aftermath of the Vietnam War (South, 1985), periods of unemployment (South, 1985) or economic stress (Masarik et al., 2016), and following Hurricane Hugo, which saw an increased divorce rate in the 24 counties that were declared disaster areas compared to the 22 other counties in the state (Cohan & Cole, 2002). It is possible that facing a serious threat can lead to the decline of the marriage due to factors including an increase in stressors such as mental health challenges (i.e., depression or PTSD) or demoralization (Norris et al., 2002), or even the challenges of meeting basic daily financial needs (Conger et al., 2010). In contrast, other studies have shown an increase in marital solidarity in the face of a crisis. In the aftermath of the terrorist attacks on September 11th, 2001, there was a decrease in divorce in the surrounding NYC and Bergen County, NJ areas (Cohan et al., 2009).

To the best of our knowledge, there are very few qualitative studies on the impact of stress on couples. In them, external stressors have been found to lead to higher levels of couple cohesiveness (Ben‐David & Lavee, 1996) as partners can instill hope, provide daily care, encourage autonomy (Waddell et al., 2020), and provide support (Donnellan et al., 2014). Levels of relationship satisfaction in the face of stress have been found to be related to more communication, emotional expressiveness, high levels of information‐sharing, and focused on conflict resolution (Nelson Goff et al., 2006, 2014; Wick & Nelson Goff, 2014).

Although there can be a seemingly ongoing conflict between spouses vis à vis work and home domains (French et al., 2018), research has found that the spouse can also serve as an extraordinary source of support (Hilpert et al., 2018). Dyadic coping can result in the stressed partner feeling cared for and understood by the other, as well as in a buffer effect, wherein one partner buffers the other from the negative effects of the stress (Falconier et al., 2013; Merz et al., 2014). Given the clear interrelatedness of spouses, in the current study we wished to apply and extend the FSM to frontline workers and their spouses as they navigated the COVID‐19 crisis.

The coronavirus pandemic has been a historic, black swan event (Halliburton, 2020) that has resulted in acute and chronic stressors for those working on the frontlines of the pandemic, as well as for their partners. This study focused on the acute crisis period that frontline doctors and nurses and their partners encountered during the first 3 months of the COVID‐19 outbreak at hospitals in NYC. Given that there is a paucity of research on the experiences of partners of frontline workers, and that the FSM highlights the potentially negative effect of stress on couples over time, in this study we aimed to learn:

a. How did frontliner partners deal with stress under the acute threat of the COVID‐19 outbreak?

b. How did this acute crisis impact the couples' relationship functioning?

## METHOD

### Participants

This study was conducted among 29 spouses or romantic partners of doctors and nurses who had been working on the frontlines with COVID‐19 patients in NYC hospitals between February 29 and June 1, 2020. Of the frontline workers, 22 were doctors, and seven were nurses. The frontliners worked in medical units including the emergency room, obstetrics, nephrology, pulmonology, rheumatology, gastroenterology, internal medicine, and rehabilitation medicine. Several of the frontliners were reassigned by their hospital administration to COVID‐19 wards from pediatric or surgery departments and had little previous experience working with adults or respiratory issues. Interviews were ceased when there was enough variability in spouses' personal backgrounds, the data collected reached saturation, and no new information emerged (Hennink et al., 2017).

Although 25 married spouses and four cohabiting partners were interviewed, for the sake of simplicity they will often be described in the text as a “partner.” Of the partners interviewed, 12 identified as female and 17 identified as male. Partner ages ranged from 29 to 71 years (*M* = 38.8). The couples had been together between 3 and 45 years (*M* = 13.5). Twenty couples had children together, averaging one‐to‐two children per family. Twenty‐seven couples identified as heterosexual, and two couples identified as homosexual. Racially, 24 identified as Caucasian or Jewish, three were Asian, and two were Latina. None of the partners were frontline workers; their professions included but were not limited to dentistry, education, psychology, social work, law, technology, childcare, music, and entertainment. Four of the partners had been sick with COVID‐19 early in the pandemic, two of whom believed they had contracted the virus as a result of their partner's work. One partner was widowed after losing her husband to COVID‐19 at the start of the pandemic, which he contracted at the hospital Table 1.

**TABLE 1:** Participant demographic information

Pseudonym	Gender	FrGender	Age	FrAge	Dr/Nurse	Years together	Married	Children?	Number of children	COVID+
Abby	F	M	32	34	Dr	10	N	N	0	
Adam	M	F	33	32	Nurse	7.5	Y	Y	2	
Andrea	F	M	33	32	Dr	8	Y	Y	1	
Caroline	F	M	42	48	Dr	20	Y	Y	2	
Eli	M	F	36	36	Dr	8	Y	Y	1	
Frida	F	M	34	39	Dr	13	Y	Y	3	
Gabriel	M	F	33	33	Dr	8	Y	Y	1	
Howard	M	M	42	42	Nurse	15	Y	N	0	Y
Isadore	M	F	41	41	Nurse	18	Y	Y	2	
Isaac	M	F	34	33	Dr	4	N	N	0	
Jacob	M	F	46	37	Dr	18	Y	Y	3	
Jonah	M	F	31	32	Dr	3	N	N	0	
Josh	M	F	41	39	Dr	17	Y	Y	3	
Leon	M	M	39	33	Dr	4	N	N	0	
Leora	F	M	31	31	Dr	9	Y	Y	1	
Matthew	M	F	33	28	Dr	8	Y	N	0	
Marina	F	M	34	35	Dr	6	Y	Y	1	
Megan	F	M	61	58	Nurse	36	Y	Y	2	
Michael	M	F	34	31	Dr	13	Y	Y	1	
Nicole	F	M	68	68	Dr	45	Y	Y	3	
Nina	F	M	32	35	Dr	9	Y	Y	2	
Paul	M	F	71	72	Dr	44	Y	Y	3	
Rachel	F	M	32	34	Nurse	5	Y	N	0	Y
Ron	M	F	38	40	Nurse	10	Y	Y	1	
Seth	M	F	40	31	Dr	10	Y	Y	1	
Sam	M	F	40	38	Nurse	18	Y	Y	2	
Samantha	F	M	29	29	Dr	15	Y	N	0	Y
Steve	M	F	34	35	Dr	6	Y	Y	1	Y
Talia	F	M	31	30	Dr	6	Y	Y	1	
*Range*			*29–71*	*28–72*		*3–45*			*0–3*	
*Mean*			*38.8*	*38.1*		*13.5*			*1.3*	

*Note*: Words preceded by “Fr” stand for “Frontliner.”

#### Ethical considerations

All participants agreed to voluntarily participate in the study, and signed consent forms. The partners were given a brief explanation of the study's aims. All identifying information about the partners, the frontliners, and places of employment were changed or omitted. At the end of the informed consent, partners were provided with the contact information of professionals, in the event they needed support for emotional distress after the interview. Research questions were approved by the [redacted] institutional review board.

### Procedure

Participants were recruited via snowball sampling. In order to be eligible for the study, partners needed to be at least 25‐years‐old, fluent in English, have a spouse or partner who was a doctor or nurse treating COVID‐19 patients in NYC hospitals, and were not themselves frontliners treating COVID‐19 patients. The partners had to be in a relationship with the frontliner during the period of the outbreak. Fliers were posted on Facebook and LinkedIn, starting in June 2020, and were also sent to various medical associations and medical departments in the NYC area. The first author's email address was included in the flier, and participants reached out to her if interested. Consent forms were sent to participants via email, which they read, signed, and returned electronically to the first author. None of the participants in the study were previously known to either of the two study authors. Seven participants referred an additional one‐to‐two other participants to the study. Otherwise, the remaining participants were respondents to the fliers that were posted online.

Data were then gathered via semi‐structured interviews that were conducted over Zoom or telephone, and recorded. All interviews were conducted in English by the first author and lasted between 30 and 50 min. The NYC COVID‐19 pandemic broke out on February 29 and continued until June 1, 2020, and the interviews took place between June 29 and October 2, 2020. At the time of the interviews, all participants who had been living separately from their partners during the outbreak had resumed living together. The interviews were transcribed by the first author or one of four university students who assisted with the transcriptions. All participants were asked the same questions. There were no rules of interaction for the interviews, and the overall tone of the interview was one of warmth and respect. The participants were assured of confidentiality and were asked to speak freely of their experiences. Thus, interviewees could, for example, express negative, critical, or resentful comments about their spouse or the relationship.

The questions posed to participants included (but were not limited to) questions about levels of stress, coping, and threat of infection. For example: *What has been your experience during the COVID‐19 pandemic? In what way, if any, has your partner's job impacted your daily life since the pandemic began? What challenges have you faced throughout this pandemic? How do you think the pandemic has impacted your relationship? When you look at the future of the pandemic, what do you think will happen to your partner and your relationship going forward?*

The researchers used thematic content analysis (Braun & Clarke, 2006) to analyze the data. This type of analysis involves three steps: (1) coding, which consists of dividing the material using a coding grid based on dimensions drawn from the scientific literature and the empirical material, while allowing the emergence of new codes that are data‐driven; (2) categorizing, during which all the codified extracts are collated in order to make sense of the narratives and to create subthemes; and (3) linking, which involves identifying links between the subthemes to establish major themes. In the current research, the first author read all the transcripts thoroughly and performed open coding, marked separate content units of meaning arising from the interviews, and shared these materials with the second author. These units of meaning were discussed several times between the authors, so as to ensure that the content raised was well represented and that the theme titles and sub‐categories represented the text. The authors together analyzed the findings, discussing them until agreement regarding the content of each subtheme was reached. The third step of linking was conducted together, and the authors identified the links between the subthemes by creating the three themes (e.g., combining types of support into one theme).

The first author is a clinical psychologist with a specialty in trauma. She works both as an instructor at a university and as a clinician in private practice. She previously worked as a postdoctoral fellow for the second author, and they have written multiple papers together. The second author has extensive experience in trauma research, specifically on the effects of trauma on family relations. The interview and the analysis were based on the literature, the authors' earlier experience in trauma research, and their drive to understand the unique challenges presented during the pandemic.

## FINDINGS

Data analysis resulted in three main themes, each with subthemes:

1. “The burden of running the home independently” refers to the enormous stress that was placed on many partners, with duties including sole childcare, food preparation, and reconfiguring their professional responsibilities in support of their partner.
2. Support provided for the frontliner included the following categories: (1) concrete support, (2) emotional support for those on the “frontlines of war”; (3) sexual and physical support via abstention.
3. Effects of the pandemic on the relationship:

- “Writing a will and discussing the possibility of death” refers to challenging conversations that partners had with one another as they faced the real possibility of illness or death should they contract the virus.
- “No relationship‐ending threats; instead, pride and support” focused on how, when partners came to confront unforeseen stress and household responsibilities, there were no threats made to end the relationship.
- “A strengthened relationship” refers to how the stressful times they endured together resulted in a strengthening of their couplehood.

### Theme 1: The burden of running the home independently

Frontliners were exhausted from working long shifts that were physically and emotionally draining (Goldstein & Weiser, 2020), as well as from working extra shifts due to understaffing, as so many coworkers were unable to work due to being ill with COVID‐19 (Ramachandran et al., 2020). An enormous burden was therefore placed on many partners, with duties including balancing solo childcare, household tasks (i.e., cooking, cleaning, laundry, etc.), and reconfiguring their own professional responsibilities in support of their partner. Partners were often drained and exhausted as well, due to independently managing a host of responsibilities. Nina, who was home with a toddler and newborn, shared the following: “*So I was already alone with the kids during the day, and then it was like nighttime too, and I was an absolute wreck*. … *that was, you know, definitely like the low point for me…. It was literally 24/7 me with the kids*.” Out of fear of a family member or paid help contracting COVID‐19 as a result of the frontliner's work, many spouses had to do everything solo. Seth, who could no longer call on extended family support for his newborn, said: “*The biggest shift for me was that we no longer were getting any help with our little one. We were quarantined and I was working from home and also with a baby full time with no help. No one was coming*.” For the couples without children or with adult children who lived elsewhere, the burden shouldered by the spouse was considerable. Jonah said, “*I tried to keep the apartment as clean as possible for sure. Like I was trying to do as many tasks and things like that as much as possible*.” When speaking of her husband's role in the hospital, Caroline shared, “*His job takes precedence over everything now*.”

Partners spoke of both the joys and drawbacks of navigating the pandemic outbreak with children. Josh, the father of three young children, said about his experience of trying to work from home, “*We don't have normal independent time, and there would be kids barging in while we were on work calls*.” The children would also be confused as to “*why Mommy was home, but not available*.” There was a fear of getting infected, specifically if both parents were to become sick with COVID‐19, and “*then we will both go down and no one will take care of our kids*.” At the same time, Steve, who cared for his small daughter full‐time, said, “*The structure that having a two‐year‐old around gives you has made my life feel very normal. It's been really nice having her and having something to do all the time*.” The challenges and structure that young children provided stood in contrast to the situation of the eight couples in this study without children.

Partners also experienced social isolation. Whereas in pre‐pandemic times partners could turn to friends and neighbors for assistance, now these supports kept their distance out of fear of contagion. As Marina said: “*[My husband's] line of work definitely complicates social interactions. At the same time, I feel like I understand why we have to stay separate from people… I see us as a tainted family even though my husband has been very careful with PPE and hasn't been infected*.” Friends also stayed away from the frontliner families, as Jacob shared: “*We have been treated differently by some of the people we know because of what my wife does for a living… the kids and the parents who, who would otherwise feel free to hang out with us, have been a little more hesitant*.”

Fearing for the health and safety of their loved one, plus balancing household responsibilities, led to a consistent theme for the partners, as Michael voiced: “*I don't think I've ever had such a stressful time in my life*.” The stress was due to a variety of factors. In Andrea's words: “*It's been one of the highest stress times of my entire life, if not the most. Very high anxiety, feeling really preoccupied, having to accept that there are factors outside of my control that are dictating how we live our lives, and worrying about health and safety of my loved one*.”

In addition to independently running the home, the partners also provided concrete, emotional, and physical support to the frontliner as they navigated the outbreak of the pandemic.

### Theme 2a: Concrete support

In addition to emotional support and management of household tasks, partners provided concrete support in personally sourcing PPE for the frontliner when there were insufficient resources available at their work. It was clear that without PPE, the frontliners were at risk for soon becoming patients themselves. Samantha recalled, “*Then also trying to find masks, because there was a point at which the hospital was unable to provide proper PPE, so then it was up to me to try and find those materials for him, whether it be through friends, or family… it kind of became just how to best keep a safe space for him… we actually had family members send us masks*.” Matthew grew resourceful in his quest to protect his wife: “*We sourced our own protective equipment from Amazon and E‐Bay*.” Partners did not try to prevent their frontliners from going to work; rather, these partners were so supportive that they located the necessary materials to keep their beloved ones safe.

Partners did not feel they could rely on the frontline worker's hospital, the local or state government, or even their spouse's union for support. Abby said angrily, “*There was even a time when the hospital administration was saying* [to the doctors and nurses], ‘*This is your own responsibility to take care of your health and not to wear masks because it scares the patient’, and any result in defying this rule of not wearing masks would end in termination or a sort of reprimand*.”

Not all partners had the opportunity to provide concrete support. Megan, a widow who lost her husband to COVID‐19 at the start of the pandemic—likely due to his lack of PPE on the job—sobbed as she spoke of her late husband's hospital supervisor's betrayal, as well as that of his union representatives. When her husband was sick and later died of the virus, no one from his workplace or union reached out to Megan to offer condolences or support of any kind: “*No one even called to ask how he was… They said that because of HIPAA* [Health Insurance Portability and Accountability Act of 1996, a federal law that protects information about a patient's health from being disclosed without the patient's consent], *nobody is allowed to ask anything. I was so pissed, and said, ‘I also work in a hospital, I know what HIPAA is, and to ask how a co‐worker is doing is not HIPAA’ and then I said, ‘Thanks for your lack of empathy’. I was just so pissed that neither the supervisor nor anyone else asked how he was doing. I felt so bad, and I was just crying all day*.”

Oftentimes the spouses were the last line of defense, as other organizations that could previously have been relied upon were not there to support or protect workers during this crisis.

### Theme 2b: Emotional support for those on the “frontlines of war”

The partners provided significant emotional support to the frontliners. Although their frontliners were often away from home on long shifts, the partners would later hear of the horrors they endured. Isadore remembered: “*She would go to the hospital and she'd just be in these rooms with all these patients on ventilators, who she was doing everything to save, even though there was a strong chance, just statistically, they were going to die. So, it felt truly apocalyptic, and I think she used phrases like ‘war zone’… Harrowing. Apocalyptic*.”

Their partners' experience was so physically and emotionally all‐consuming that many of the partners used metaphors of war to describe their frontliner's experience. Frida said, “*I thought a lot about how the wartime analogy was apt, except for the part where soldiers voluntarily sign up, they know what they are potentially getting into, so there are no surprises, like big‐picture surprises, like the day‐to‐day of being in the military, things can be sprung on you, but you kind of know that you are signing up to put your life on the line*.”

Partners shared their experience of what it was like to live with frontliners who were afraid and overwhelmed. Frida said: “*He was the most anxious, nervous, overwhelmed I have ever seen him. He's the prototypical surgeon … very cool, calm, and collected, not a super emotional person, very cool under pressure, and this was like, he had unbelievable anxiety*.” The partners noted how the frontliner's mood had changed greatly as a result of their work. As Nicole said: “*I have never seen him so disheartened. And we've been married for 45 years. I have never seen him so despondent and so helpless*.”

Yet in the face of extreme stress and anxiety, partners uniformly attempted to offer their frontliners emotional support. Steve said, “*I just thought about the fact that I have to be supportive… It's really a level of selflessness that I couldn't understand before. But then once I did, and knowing that my job was to support her and support the family in this way that I was able to, then I knew that this was what I had to keep doing*.”

### Theme 2c: Sexual and physical support via abstention

Partners willingly abstained from physical touch and sexual relations, and sometimes lived separately to avoid contagion; nevertheless, they still provided emotional and practical support to the frontliners. Josh was home with their three young children while his wife worked extended hours at the hospital. When reflecting on this crisis period, Josh focused on a particular area of difficulty: “*There were also physical barriers. We also couldn't even kiss each other. Things like that, I never thought much about, because it was natural. You do it when you say goodbye, in the morning, you kiss each other whenever, throughout the day. But since it wasn't happening, it was even more magnified*.”

The lack of touch reinforced for the partners how their lives had drastically changed, leaving them feeling stripped of normalcy within their couplehood. Jacob said, “*In March and April, we weren't sharing food, we weren't sipping each other's coffee, all of these things that you instinctively do as a couple, leaning in for a kiss, you just don't, you second‐guess yourself, ‘Are we doing that yet? Nope, we're not doing that yet’*.”

In addition to not touching, some couples chose to physically separate during the outbreak. Reasons included partners having a pre‐existing condition that placed them in an especially high‐risk group, pregnancy, or wishing to keep newborns or young children safe from possible contagion. Andrea opted to move in with her parents with her newborn: “*The whole point of us living separately was so I could stay healthy to take care of the baby… I did return to our apartment twice … and during those times we both wore masks and gloves and stayed six feet apart and spent very limited time in the apartment at the same time together*.”

Living separately also provided the opportunity for the frontliners to focus exclusively on their work and not worry about infecting their partners, running a household, or caring for family members. As Leora explained, “*He would be in the city for the days that he would be working, like multiple days in a row, he'd stay in the city, commute to work and live his own life. When he had two or more days off, he'd come out here, and he would sleep in a separate room, on a separate, different side of the house*.”

When living separately, frontliners would sometimes come to visit their partner and children from a distance. However, as Frida shared, a sense of fear accompanied seeing her husband: “*Among the most difficult parts of this whole thing was that the risk of infection was really real. He felt very toxic. We were thrilled that he was coming back to visit, and I was beside myself with excitement and happiness to see him, and my kids were beyond thrilled. But I had this nagging fear in my mind of his physical presence*.”

There was a financial component to the choice or ability to separate; income played a significant role in the ability to social distance (DeLuca et al., 2021). For instance, some of the doctors were just finishing residency or fellowship, and were living on a tight budget. Social class issues and availability of economic resources became apparent in the type of living space available to frontliners and their partners. For example, limited budgets resulted in some frontliners living with their partners in small studios, precluding the option of social distancing. Other doctors and nurses were at the beginning of their careers and had limited savings and resources, so that living separately was not always feasible. Abby, whose fiancé was at an early point in his career as an ER doctor, shared how she became “*concerned about how to keep everything as sterile and safe as possible, but in a 600‐foot apartment that's pretty impossible… We didn't have the luxury of having space so that we could*.” In contrast, other frontliners were advanced in their careers, and had the resources to live separately. Some couples had family support, which could make a significant difference. Matthew said, “*During most of that period my wife stayed at another apartment owned by family that was vacant in the city. She stayed there to keep me protected, to keep me safe*.”

### Theme 3a: Writing a will and discussing the possibility of death

A frightening aspect of the pandemic's outbreak was the growing number of doctors and nurses who were falling ill or dying from the virus due to on‐the‐job exposure. Partners reflected on conversations regarding last wills and testaments for the first time, asking their frontliners for specific directives should they pass away. Frida said:

He would make a lot of jokes. His way of handling it was by calling me and being like, “Well, if we never talk again, because I die tomorrow because I get this virus, and deteriorate rapidly and get put on a ventilator” … We gave each other passwords to things, we made a Google doc where we organized some stuff, just so we were a little more organized in case crazy things happened. We talked about the worst happening and putting some things in order, making plans for what hospital did he want to go to if he woke up and had COVID‐19, things like that.

Partners suddenly became aware of how little they knew of their financial situations, or of how they would manage if their loved one passed away prematurely. Prior to the pandemic, working as a doctor or nurse was not considered to be a high‐risk profession (Sauter & Stockdale, 2019). Partners were stunned to realize what a new and very real risk their partners were exposed to now. Partners also accepted that their frontliner was opting to work, even with the ever‐present threat of contagion. As Abby explained:

At first, I was terrified. But we had a conversation, and it was a kind of surreal, morbid conversation, in which he told me that this was his duty and his passion and his purpose in life to serve and help and protect the communities, and that if he were to die it would be in the way he would have wanted to live his life. So that was a really hard conversation to kind of take in. And of course, very emotional because I couldn't even fathom that we were even having the conversation or needed to have the conversation.

It should be said that despite the extremely high stakes of working on the frontlines, not a single partner interviewed dissuaded their frontliner from going into work. Instead, they had conversations about the possibility of death.

### Theme 3b: No relationship‐ending threats; instead, pride and support

Several of the partners spoke about stress, tension, or bickering in the relationship, saying, “*It has been a rough year for our relationship as well*.” However, none of the partners left or threatened to leave the frontliner. Although there was no pre‐study assessment for levels of cohesion or stability, all couples shared how despite the stresses and challenges they decided to stay together. As Leon said:

With stress levels, a lot of the ups and downs probably come from emotional management with my partner. It tests our relationship when we're both at home … it's a little stressful to manage and find your own personal space in this 650‐square‐foot apartment. I get stressed out when I'm trying to help him find comfort when he comes home from being at the hospital.

Yet when Leon had the chance to leave his partner and move in with his mother he said, “*But I was like*, ‘*My man is on the front line, I should probably stay here and comfort him*’.”

Almost all partners shared how proud they felt of their frontliner, and their desire to be supportive. Leon said, “*I was saying I'm not gonna, like, go home and leave you*—*I'm going to be here and support you throughout the entire thing and continue to support you as best I can*.” Steve said, “*I think I just thought about the fact that I have to be supportive, my job was to support her and support the family in this way that I can, this is what I have to keep doing*.”

And Seth said, “*I felt proud, I wanted to be supportive, just continue to be a calming even‐keeled presence. I felt concerned, but proud at the same time. Hers was one of the few professions right now that was of service. Her profession just felt so important*.” Support became a top priority. Seth said, “*I basically made my job over the last six months to support her as best I could and make sure she has everything she needs so she doesn't have to worry about what is going on at home…. I just thought about the fact that I have to be supportive*.”

### Theme 3c: A strengthened relationship

Many partners said that the outbreak had strengthened their bond. Andrea mused, “*I think it's made me appreciate every second now that I have with him… I think it's made me admire how selfless he was in his sacrifice. So, it's definitely strengthened our relationship*.” Michael echoed: “*I'm of the mind that it made us stronger and that it's given us a deeper reserve to call upon when we're challenged. And I think we're better for it, especially also seeing how brave my wife was when push came to shove. It has all left us in a better place in terms of our relationship… We've gotten to know each other a lot better than we did, even though we were together for a long time before this happened*….”

The crisis also made the partners closer, as Talia explained:

I think it's made us more closely dependent on one another. I think being apart made us realize how much of a family unit we already were. During the peak of it, when he was really treating a lot of very sick COVID‐19 patients, I felt like I was his emotional outlet. He would end the day walking home from the hospital and call me to sort of unload what had happened during the day… we really shifted to being our own family unit and being so dependent on one another, in a way that we hadn't before.

The pandemic also led to a re‐prioritizing of values and mindsets. As Sam explained, “*It is a reminder that money is not important. I think what you have is the family relationship. It actually makes us prioritize what is important out there*.” Abby said, “*It's definitely given us both a perspective on how fragile life is, it has given us a new appreciation for how much time we have and how to make the most of that time together*.” The trauma of what the partners experienced led to growth. In Jacob's words: “*I frankly trust her more now as a result of that because I know that she does go through hell, and can explain it, and can come back from it*.” Not only did the pandemic outbreak not threaten the couple relationship, but it led to the growth of a stronger relationship and to loyalty and support in the face of intense stress.

## DISCUSSION

In this study, we aimed to learn from the point of view of the partner about how frontliner couples dealt with stress under the acute threat of the COVID‐19 outbreak and how this crisis impacted their relationship functioning. Partners clearly faced enormous stressors, burdens, and dilemmas in a range of areas. Two should be specifically mentioned. First, being in close contact with the frontliner could have been life‐threatening for the whole family. Therefore, despite their wish to be together, couples had to keep a distance from one another, as well as socially distance from neighbors, friends, and family. At the same time, partners were simultaneously often navigating who they should protect, and to what extent: themselves, the frontliners, children, friends, community, or extended family. Partners had to navigate the spillover of needs and responsibilities from one domain to another (Dekel et al., 2016).

Second, watching their partners being called to the frontlines, where they were not receiving the necessary protection, raised an ethical dilemma as to whether or not to support the frontliner continuing to work. Additionally, some partners felt estranged from, let down by, or even enraged by the lack of formal institutional support, including insufficient PPE as well as the absence of institutional support during these times of severe stress and death. Findings were similar to those from a qualitative study that assessed participants' feelings of trust toward political and social systems in the aftermath of Hurricane Katrina, revealing that “lack of competency” among all governmental officials and workers was the most frequently marked answer in the category of trust (Cordasco et al., 2007). Although earlier studies have recognized workers' ethical dilemmas during times of shared trauma, our study brings to light additional ethical dilemmas for spouses, the whole family system, and the broader hospital system in this unique shared trauma.

We identified a few themes of partners' support to their frontline workers. Some, such as emotional support, are already known in the literature. However, given the definition of support as “verbal and nonverbal behavior produced with the intention of providing assistance to others perceived as needing that aid” (MacGeorge et al., 2011, p. 317), our study adds a unique element, pointing to the partners' acceptance *of absence* of physical and sexual closeness as one such manifestation of support during these times. Although studies on couples who live remotely or serve in the military have addressed the challenges of support in military families (Griffith, 2020), the current situation created a unique challenge of providing support via the absence of behavior. In a study conducted on physical touch for 585 partnered adults during the pandemic, more physical distancing was associated directly with *lower* psychological distress and *better* relationship quality; indeed, for those who were cohabiting in satisfying romantic relationships, the physical distancing appeared to facilitate relationship‐positive behaviors (Burleson et al., 2021).

Two additional subthemes emerged when we looked at the effects of this crisis on the relationships. First, couples made specific plans in the event something might happen. Second, in line with earlier studies (i.e., Cohan et al., 2009; Deryugina et al., 2014), here too we found an increase in relationship solidarity in the face of a crisis and an absence of relationship‐ending threats. There could be several possible explanations for these findings. First, the threat of the pandemic was unambiguous and acute: conditions which can be unifying for a couple. Conversely, high ambiguity can challenge relationships within a family and can make cooperation even more challenging (Boss, 1987). Here, the couples were not only facing a threat to their couplehood, but also were together in navigating a threat to the city in which they lived, as well as a global danger. More specifically, the couples were navigating the external threat of a pandemic, as opposed to an internal threat to their relationship (i.e., infidelity or conflict). Therefore, rather than being a source of stress spillover in their relationship or family, the external threat here may have unified the couple (Merz et al., 2014; Neff & Karney, 2004). Some studies have found that crises decrease relationship stability (Masarik et al., 2016; South, 1985), and others have found that they increase stability (Cohan & Cole, 2002). In the current study, the crisis conditions of the pandemic outbreak were uniquely all‐consuming and acute in its outbreak and served to strengthen relationships and enhance the cohesion between the partners.

Additionally, stress theory predicts that spouses can have a buffering effect on one another in the face of a crisis or even a challenge, via providing social, emotional, and logistical support (Stroebe et al., 1996). As we wrote earlier about support, each partner may have buffered the other from stress, a finding that bolsters the FSM. As seen in this study, and in support of the FSM, the external stressor of a pandemic outbreak seems to have led to high levels of couple cohesiveness in the couples examined here (Ben‐David & Lavee, 1996), as well as more communication, information‐sharing, and low levels of conflict (Nelson Goff et al., 2006, 2014; Wick & Nelson Goff, 2014).

For some couples, partners lost their jobs due to the pandemic but still had financial security because of the frontliner's hospital wages and job security. Although it is possible that there are frontliner couples who stayed together for pragmatic or instrumental reasons (i.e., economic, material, spatial, cultural, social, relational, etc.), in the current study, seven of the partners who lost or could not find jobs due to the pandemic (i.e., musicians, bartenders, recent graduates of master's programs etc.) took on new roles within the couple. For example, for the couples with children, the partner took on the much‐needed role of full‐time caretaker. In childless couples, the partners focused on cooking or other household tasks. All partners who faced employment challenges spoke of providing unpaid labor (Hazarika & Das, 2021), such as maintaining social connections, completing chores, and providing emotional support. In line with the FSM, the couples here demonstrated support for one another in the face of the external stressors of the pandemic outbreak; they showed sensitivity, concern, and problem‐solving as the frontliner contended with the stress of the outbreak (Conger et al., 1999). In the face of a crisis, the partners provided daily care, provided hope and encouragement (Waddell et al., 2020), and provided support across a variety of domains.

Finally, the partners here also navigated a shared trauma together with their frontliner (Dekel & Baum, 2010). Shared trauma occurs when professionals both live and work in the same community as the people they serve, so that they are exposed to and threatened by the very same disaster or collective trauma as their clients (Dekel & Baum, 2010; Tosone, Nuttman‐Shwartz, & Stephens, 2012). The majority of studies on shared trauma have focused on mental health professionals, and have highlighted the fact that therapists are exposed to this collective trauma as private citizens and members of a family and also as professionals who are aiming to treat their patients in the aftermath of an event (Tosone et al., 2011; Tosone et al., 2015). In this study both the frontliner and the partner were exposed to a global crisis. Professionals in such situations have been found to be at risk for emotional distress both during and in the immediate aftermath of the crisis, with effects including burnout, insecurity about their professional abilities, and guilt due to a shared trauma overload (Tosone, Nuttman‐Shwartz, & Stephens, 2012). In addition, participants were found to have experienced conflicts of loyalty in choosing between their professional obligations to patients and their needs or wishes to remain beside family members and loved ones during an emergency (Dekel et al., 2016). Previous research on shared trauma found that “the family domain was the vulnerable space for the participants” and that workers found it difficult to function in their professional roles if they were not sure their own family members were safe (Dekel & Nuttman‐Shwartz, 2014, p. 235). In light of these findings, follow‐ups must be conducted with medical teams.

### Limitations

The couples in this study were assessed in the months immediately after the pandemic's outbreak. As such, it would be of interest to see the chronic effects of stress over time on their relationships. Research suggests that ongoing stressors are more detrimental to health and well‐being than are short‐term or occasional events (Lepore et al., 1997). Indeed, chronic stress can have negative effects on an individual's health, emotions, and immune and nervous systems (Kemeny, 2003). Second, the sample focused on frontline doctors and nurses in a specific geographic area: NYC. Future studies should examine couples in other geographic locations. Third, although significant attempts were made to recruit participants from diverse backgrounds, the majority of the sample self‐identified as Caucasian and heterosexual. More research is needed on the experiences of couples from other backgrounds (i.e., socioeconomic, racial, additional couples from the LGBTQ community, etc.) It would also be of interest to interview non‐cohabiting partners of frontline healthcare workers (especially ones who were not in well‐established relationships) or the partners of frontline hospital workers who were exposed to COVID‐19 but were not doctors and nurses (e.g., janitors, food preparers, etc.). Fourth, as this study was conducted from the partners' points of view, it would be of value to interview the frontliners about the role they saw their partners assume as they battled the pandemic. Finally, many of the couples examined here chose to stay together throughout the stresses and strains of residency, and reported high levels of commitment. It is also possible that higher levels of relationship cohesion were due to the participation of couples who opted to remain together—rather than break up. It would be interesting in future studies to compare the experiences of couples whose frontliners were at an early stage in their medical careers versus couples whose frontliners were at later stages of their careers, and also to interview couples who ended their relationship during the outbreak.

From a clinical perspective, mental health professionals may be asked to provide support to partners or couples in times of acute stress. As can be seen here, the spouses and partners of those working on the frontlines are a stressed population. They carry an extensive caregiving burden (Dekel, Solomon, & Bleich, 2005) and stress, and attention must be given to their unique needs as they continue to navigate the pandemic. It has been found that chronic stress can have a negative effect on relationship satisfaction over time (Karney & Bradbury, 1995; Neff & Karney, 2004). Moreover, given frontliners' ethical dilemmas during this pandemic (Hossain & Clatty, 2021), it is important to assess how partners cope together. Clinicians may wish to apply findings from studies on military couples where one partner was deployed to frontliners couples, taking note of how such couples navigate the stress of separation. Similar to the partners in this study, the partners of deployed soldiers also have non‐stop responsibilities (Wilson & Murray, 2016), relationship distress, mental health challenges (Blow et al., 2017), and difficulties in communication (Knobloch et al., 2018). Knowledge from research on partners of frontliners regarding the protective role of communication, role flexibility, emotion regulation, problem‐solving, and co‐constructing meaning (Larsen et al., 2015) could be applied and extrapolated to other couples who, for reasons not in their control, must separate.

Interventions might include addressing partners' potential secondary traumatic stress, seeing the couple together for therapy, and providing space for partners to process their experiences. However, as can be seen here, stress—while challenging and frightening—can also have a unifying effect on couples. Insofar as the pandemic is not yet over, it is possible that couples may need additional support as they navigate the chronic stresses of the frontliner's work. However, it is also important to consider the vital and potentially positive role that partners can play in supporting one another as they navigate an acute crisis together.
